# LB11. Preliminary Findings from a HIV Self-Testing Program among People Who Use Drugs

**DOI:** 10.1093/ofid/ofab466.1647

**Published:** 2021-12-04

**Authors:** Michelle Rose, Laura Guy, Steve Shamblen, Greg Guest, Adam Gilbertson, Nicholas Peiper

**Affiliations:** 1 Norton Healthcare, Louisville, KY; 2 Pacific Institute for Research and Evaluation, Louisville, Kentucky; 3 University of Louisville, Louisville, Kentucky

## Abstract

**Background:**

People who use drugs (PWUD) remain at significantly high risk for HIV infection. It is estimated that the majority of all new HIV infections are through injection drug use, with an estimated 2,500 new infections occurring annually among people who inject drugs. Although new HIV infections have been quickly rising over the past year, the Center for Disease Control and Prevention (CDC) preliminarily reported a 50% to 70% intra-pandemic decline in HIV testing. Within Kentucky, an ultra-high-risk state, multiple health departments reported all HIV testing stopped during the early stages of the COVID-19 pandemic (March-July 2020). Once testing resumed, appointments were sparse.

**Methods:**

To address low rates of HIV testing among PWUD, we evaluated the acceptability of a HIV self-testing program (OraQuick In-Home HIV Test by OraSure Technologies, Inc.) implemented at a health department in Louisville, Kentucky that services PWUD. Descriptive statistics were calculated for testing location, testing self-efficacy, reasons and motivations, ease of use, and preferences for future services.

**Results:**

From May to June 2021, a total of 230 PWUD engaged with the program (average of 18 per day). Most participants (87.8%) self-tested at the health department with the help of study staff, while the other 12.2% tested at home and returned at a later time. Approximately 77% of participants reported the self-test kit made them feel better able to keep track of their HIV status compared to standard testing methods. The most common reasons for testing were wanting to know their status (85%), the test was free (37%), fast results (31%), more privacy (23%), and recent high-risk drug use and sexual behaviors (17%). Virtually all (97%) reported the test kits were very easy to use. For future availability of self-test kits through the health department, 33% reported they would use them monthly, 28% every three months, 22% every six months, and 17% annually. In terms of preference for future testing modality, 72% indicated a preference for taking the kits home, while the other 28% indicated a desire to test at the health department with help from staff.

**Conclusion:**

Program participants found the self-test kits to be acceptable and easy to use. Implications for program implementation and future research will be discussed.

**Disclosures:**

**Michelle Rose, MBA**, **Gilead Sciences Inc.** (Grant/Research Support) **Laura Guy, BS, CCRC**, **GILEAD Sciences** (Grant/Research Support, Research Grant or Support)

